# Healthcare utilization and costs among intracranial meningioma patients during long-term follow-up

**DOI:** 10.1007/s11060-022-04223-0

**Published:** 2023-01-10

**Authors:** Kevin A. Huynh, Eva C. Coopmans, Amir H. Zamanipoor Najafabadi, Linda Dirven, Saskia M. Peerdeman, Nienke R. Biermasz, Marco J. T. Verstegen, Wouter R. van Furth, the Dutch Meningioma Consortium, Florien W. Boele, Martin Klein, Johan Koekkoek, Frank Lagerwaard, Pim B. van der Meer, Martin J. B. Taphoorn, Wouter A. Moojen, Jaap C. Reijneveld

**Affiliations:** 1 Division of Endocrinology, Department of Medicine, Leiden University Medical Center, Albinusdreef 2, 2333 ZA Leiden, The Netherlands; 2 Center for Endocrine Tumors Leiden (CETL), Center for Pituitary Care, Leiden University Medical Center, Leiden, The Netherlands; 3 Department of Neurosurgery, University Neurosurgical Center Holland, Haaglanden Medical Center, and the Hague Teaching Hospitals, Leiden University Medical Center, Leiden, The Netherlands; 4 Department of Neurology, Leiden University Medical Center, Leiden, The Netherlands; 5 Department of Neurology, Haaglanden Medical Center, The Hague, The Netherlands; 6 Department of Neurosurgery, Amsterdam University Medical Centers, Location VUmc, Amsterdam, The Netherlands

**Keywords:** Meningioma, Healthcare utilization, Costs, Health-related quality of life, Value-based healthcare, Patient-reported outcome

## Abstract

**Purpose:**

Few studies have reported on healthcare utilization and costs for intracranial meningioma patients, while the tumor and its treatment profoundly affect patients’ functioning and well-being. Here we evaluated healthcare utilization and costs, including their determinants.

**Methods:**

A multicenter cross-sectional study of adult meningioma patients ≥ 5 years after intervention. Patients completed three validated patient-reported outcome measures (PROMs) assessing patients ‘functioning and wellbeing (SF-36, EORTC QLQ-BN20, and HADS) and a study-specific questionnaire assessing healthcare utilization over the previous twelve months. Healthcare costs of the twelve months prior were calculated using reported healthcare utilization ≥ 5 years after intervention by the Dutch Manual for Economic Evaluation in Healthcare. Determinants for healthcare utilization and costs were determined with regression analyses.

**Results:**

We included 190 patients with WHO grade I or II meningioma after a mean follow-up since intervention of 9.2 years (SD 4.0). The general practitioner (80.5%), physiotherapist (37.9%), and neurologist (25.4%) were visited most often by patients. Median annual healthcare costs were €871 (IQR €262–€1933). Main contributors to these costs were medication (45.8% of total costs, of which anti-seizure medication was utilized most [21.6%]), specialist care (17.7%), and physiotherapy (15.5%). Lower HRQoL was a significant determinant for higher healthcare utilization and costs.

**Conclusion:**

In patients with meningioma, medication costs constituted the largest expenditure of total healthcare costs, in particular anti-seizure medication. Particularly a lower HRQoL was a determinant for healthcare utilization and costs. A patient-specific approach aimed at improving patients’ HRQoL and needs could be beneficial in reducing disease burden and functional recovery.

**Supplementary Information:**

The online version contains supplementary material available at 10.1007/s11060-022-04223-0.

## Introduction

Meningiomas are the most prevalent primary intracranial tumor, representing 39% of all central nervous system tumors [1, 2]. Approximately 80% of patients suffer from a benign World Health Organization (WHO) grade I meningioma, which is associated with a near-normal life expectancy [2–4]. Patients present with a variety of symptoms, such as vision impairment, mental changes, and seizures [2–4].

A recent cross-sectional study reported that WHO grade I or II meningioma patients still experience a significant disease burden after a median follow-up time of nine years, despite most patients having undergone total resection and are considered cured radiologically [5]. These patients reported clinically relevant impairments in role functioning due to physical and emotional health problems, and experienced more impairments at work than healthy controls. Patients suffered more frequently from self-reported anxiety or depression, and had more neurocognitive deficits than controls [5]. It is thus plausible that patients with these impairments in functioning and well-being utilize more healthcare in comparison to the general population.

Several studies have reported the costs of different treatment modalities (e.g., surgery and sterotactical radiosurgery) for meningioma patients [6–8]. However, to date, no studies have described healthcare utilization and its related costs among meningioma patients on the long-term. This study aimed to report healthcare utilization, healthcare costs, and their determinants for these patients in long-term follow-up. Identification of disease- or care-related determinants will enhance the understanding of factors driving healthcare utilization and costs, which can be used to improve efficiency of care and may consequently improve health outcomes of meningioma patients.

## Methods

### Study design

This is a multicenter cross-sectional study on long-term disease burden in meningioma patients. Other outcomes have been described previously [5]. Meningioma patients were eligible for participation if the end of primary antitumor treatment (i.e., surgery or radiotherapy) was at least five years prior to recruitment, or in case of solely an active MRI surveillance, at least five years after diagnosis. Patients with disease recurrence during the follow-up period that needed antitumor treatment were excluded from the study. Inclusion criteria were age ≥ 18 years and histologically confirmed WHO grade I or II meningioma in case of surgery or clinical diagnosis based on MRI of meningioma in case of radiotherapy or active MRI surveillance. Exclusion criteria were a history of whole-brain radiotherapy, diagnosis of neurofibromatosis type II or any other neurodegenerative disease, and insufficient mastery of Dutch language.

Patients were recruited between July 2016 and April 2019 from neurosurgery, neurology, and radiation oncology outpatient clinics of two academic tertiary referral centers and one large non-academic teaching hospital in The Netherlands (Leiden University Medical Center, Amsterdam University Medical Center, location VUmc, and Haaglanden Medical Center). A non-responder analysis was described in a previous report, showing no large differences between patients who did and did not participate [5]. The study was approved by the Medical Ethics Committee of all participating centers (NL54866.029.15).

### Patient-reported outcome measures

Patients completed three validated patient-reported outcome measures (PROMs). Healthcare utilization was measured with a study-specific questionnaire). This questionnaire was based on the existing Treatment Inventory of Costs in Patients with psychiatric disorders (TIC-P) questionnaire. During a consensus meeting, healthcare professionals involved in the treatment of meningioma patients selected those aspects that were considered relevant for the care trajectory of meningioma patients. The study-specific questionnaire to measure healthcare utilization was used to assess the frequency of healthcare professional consultation in the twelve months prior to the study. We considered consultation with the following healthcare professionals relevant for meningioma patients: general practitioner, neurologist, neurosurgeon, oncologist, radiation oncologist, ophthalmologist, dermatologist, ear, nose and throat (ENT) specialist, endocrinologist, physiatrist, plastic surgeon, anesthesiologist, radiologist, psychologist, psychiatrist, psychotherapist and physiotherapist. Data on the reason for visitation of healthcare professionals were not collected, as these were not explicitly reported in the patient charts or questionnaire. Patients were categorized into high (≥ 3 visits) or low specialist care utilization (< 3 visits), based on the total number of visits to any relevant medical specialist during the previous twelve months. The study-specific healthcare utilization questionnaire assessed the use of medication in terms of dosage and frequency, which was used to determine the use of antiepileptic drugs, benzodiazepines, antidepressants, and hormone replacement therapy. In addition, the use of the emergency room and admission in healthcare facilities (i.e., academic hospitals, non-academic hospitals, psychotherapeutic facilities, and rehabilitation centers) was assessed. HRQoL was measured with the Short-Form Health Survey 36 (SF-36) and the European Organization for Research and Treatment of Cancer quality of life questionnaire, brain neoplasm module (EORTC QLQ-BN20). Anxiety and depression were measured with the Hospital Anxiety and Depression Scale (HADS). More detailed information about these PROMs can be found in the supplemental file (Supplemental File).

### Sociodemographic and clinical characteristics

Clinical characteristics were collected from medical records and sociodemographic characteristics were obtained via a structured interview at the beginning of the assessments. These included age at diagnosis, sex, tumor size (largest diameter at diagnosis), number of tumors, symptoms at presentation, WHO grade, and primary treatment.

Self-reported characteristics included marital status and educational level. Level of education was classified as (1) low (primary/secondary education), (2) intermediate (technical/vocational), and (3) high (academic/university) in accordance with guidelines set by the Dutch Central Bureau for Statistics (CBS) [9], based on International Standard Classification of Education: Fields of Training and Education 2013 by UNESCO [10].

### Costs

Healthcare costs were calculated from reported healthcare utilization in the study-specific questionnaire. Prices were obtained following the Dutch Manual for Economic Evaluation in Healthcare and were based on reference prices for 2016 [11]. Costs are presented as medical costs, medication costs, and overall costs. Medical costs include costs of medical specialist care, mental healthcare, admission in healthcare facilities, and emergency department visits. Medication costs are presented separately for anti-seizure medication, antidepressants, benzodiazepines, and hormone replacement therapy. The costs of primary antitumor treatment were not included in this study, since this study only included patients if the end of the antitumor treatment was at least five years prior to recruitment (i.e., patients with disease recurrence during the follow-up that needed antitumor treatment were excluded from the study).

### Statistics

Baseline characteristics, healthcare utilization, and costs were presented for the total meningioma cohort and subdivided and compared between convexity and skull base meningioma patients. Continuous variables were presented using mean with standard deviation (SD) or median with interquartile ranges (IQR), depending on distribution of the variable, and analyzed with independent t-tests or Mann–Whitney U tests, respectively**.** Categorical data were presented as frequencies with percentages and comparisons were made using Chi-squared analyses. Multivariable logistic regression analysis was used to determine associations between possible health-related determinants and high specialist care utilization (defined as ≥ 3 visits), and associations were expressed as odds ratios (ORs) with 95% confidence intervals (CIs). Associations between possible determinants and overall healthcare costs were evaluated using multivariable linear regression analysis and were presented as regression coefficients (*β*) with corresponding 95% CIs and *P*-values. Multiple multivariable regression analyses were performed to determine the association between each determinant and the outcome separately, all corrected for age and sex in order to control for confounding. Regression analyses including the HADS, SF-36, EORTC QLQ-BN20 and Charlson Comorbidity Index (CCI) scores were also corrected for educational level.

A *P*-value < 0.05 (two-sided) was considered significant for all statistical analyses. Complete case analysis was used to handle missing data, due to a low number of missing data (Supplemental File). All statistical analyses were performed using IBM SPSS 25.0 software (IBM Corp., Armonk, NY).

## Results

### Study population and patient characteristics

In total, 190 patients (n = 98, 51.6% from Leiden University Medical Center; n = 44, 23.2% from Amsterdam University Medical Center, location VUmc; n = 48, 25.3% from Haaglanden Medical Center) were included in the study. Characteristics of the study population are presented in Table 1 and have been reported previously [5]. Patients were on average 52.6 years old at diagnosis (SD 11.3) and mean follow-up time since intervention was 9.2 years (SD 4.0). The study population consisted mostly of females (n = 149, 78.4%). Tumors were located on the skull base in 93 patients (48.9%), cerebral convexity in 92 patients (48.4%), and optic nerve sheets or intraventricularly in 5 patients (2.6%). The latter group was not included in the analysis on tumor location (i.e., when comparing skull base and convexity meningioma patients). Median tumor size at diagnosis was 38.0 mm (IQR 26.0–50.0) and the majority of surgically treated meningiomas was classified as WHO grade I (n = 148, 88.6%). Most patients received surgery (n = 167, 87.9%) as primary treatment, of whom 62 suffered from any postoperative complication [i.e., cerebrospinal fluid leak (n = 8), cranial nerve deficits (n = 8), or hydrocephalus (n = 6)]. In this subgroup treated primarily with surgery, a total of 26 patients (13.7%) were treated with adjuvant radiotherapy and 13 (7.8%) with reresection. Primary radiotherapy was limited to 10 (5.3%) patients. A minority of 13 (6.8%) were solely followed with active MRI surveillance (i.e., conservative treatment). Patient characteristics stratified by tumor location are reported in the supplemental file (Supplemental File).

**Table 1:** Patient characteristics of the total group, convexity, and skull base meningioma

	Total cohort (n = 190)	Convexity meningioma patients (n = 92)	Skull base meningioma patients (n = 93)	*P*-value
Age at diagnosis (years)	52.6 (11.3)	53.5 (12.0)	51.8 (10.2)	0.302
Sex (male)	41 (21.6)	27 (29.3)	13 (14.0)	0.011*
Mean follow-up since intervention (years)	9.2 (4.0)	8.7 (4.3)	9.9 (3.6)	0.039*
Academic hospital	142 (74.7%)	67 (72.8%)	72 (77.4%)	0.470
Education level				0.893
Low	41 (21.6%)	21 (22.8%)	20 (21.5%)	
Intermediate	86 (45.3%)	41 (44.6%)	44 (47.3%)	
High	58 (30.5%)	25 (27.2%)	29 (31.2%)	
Tumor size at diagnosis (largest diameter, mm)	38.0 (26.0–50.0)	40.5 (28.5–55.0)	37.0 (25.0–48.0)	0.046*
Symptoms at presentation				
Epilepsy	31 (16.3%)	20 (21.7%)	10 (10.8%)	0.043*
Motor deficit	28 (14.7%)	19 (20.7%)	9 (9.7%)	0.037*
Sensory deficit	24 (12.6%)	7 (7.6%)	17 (18.3%)	0.031*
Visual deficit	51 (26.8%)	8 (8.7%)	39 (41.9%)	< 0.0001*
Cognitive impairment	14 (7.4%)	11 (12.0%)	3 (3.2%)	0.025*
Psychological impairment	7 (3.7%)	4 (4.3%)	3 (3.2%)	0.721
Headache	32 (16.8%)	14 (15.2%)	18 (19.4%)	0.457
Other	37 (19.5%)	16 (17.4%)	20 (21.5%)	0.480
Incidental finding	17 (8.9%)	13 (14.1%)	4 (4.3%)	0.021
WHO grade				
WHO grade I	148 (77.9%)	71 (77.2%)	76 (81.7%)	0.444
WHO grade II	12 (6.3%)	8 (8.7%)	4 (4.3%)	0.225
Primary treatment				
Surgery	167 (87.9%)	83 (90.2%)	83 (89.2%)	0.828
Radiotherapy	10 (5.3%)	1 (1.1%)	6 (6.5%)	0.118
Conservative treatment	13 (6.8%)	8 (8.7%)	4 (4.3%)	0.225
Reresection (n = 167; treated with primary surgery)	13 (6.8%)	6 (6.5%)	7 (7.5%)	0.773
Complications of primary treatment				
Surgery	62 (32.6%)	23 (25.0%)	38 (40.9%)	0.022*
No complication	122 (64.2%)	64 (69.6%)	54 (58.1%)	0.104
Simpson grade (n = 167; treated with primary surgery)				
Grade I	35 (21.0%)	23 (27.7%)	12 (14.5%)	0.036*
Grade II	57 (34.1%)	26 (31.3%)	31 (37.3%)	0.414
Grade III	16 (9.6%)	8 (9.6%)	8 (9.6%)	1.000
Grade IV	34 (20.4%)	11 (13.3%)	23 (27.7%)	0.021*
Grade V	5 (3.0%)	2 (2.4%)	3 (3.6%)	1.000
Unknown	20 (12.0%)	13 (15.7%)	6 (7.2%)	0.088
Charlson Comorbidity Index	0.0 (0.0–1.0)	0.0 (0.0–1.0)	0.0 (0.0–1.0)	0.111
SF-36				
Physical component score	50.7 (40.4–56.0)	49.8 (37.7–56.2)	50.7 (44.2–56.1)	0.421
Mental component Score	53.3 (44.9–57.5)	51.8 (40.3–58.3)	53.7 (46.3–56.9)	0.252
EORTC QLQ-BN20				
Uncertainty of future	16.7 (0.0–33.3)	16.7 (8.3–33.3)	16.7 (0.0–25.0)	0.281
Visual dysfunction	11.1 (0.0–22.2)	11.1 (0.0–33.3)	11.1 (0.0–22.2)	0.547
Motor dysfunction	0.0 (0.0–22.2)	11.1 (0.0 -22.2)	0.0 (0.0–22.2)	0.012*
Communicative deficit	11.1 (0.0–22.2)	11.1 (0.0–22.2)	11.1 (0.0–22.2)	0.644
Headaches	0.0 (0.0–33.3)	0.0 (0.0–33.3)	0.0 (0.0–33.3)	0.053
Seizures	0.0 (0.0–0.0)	0.0 (0.0–0.0)	0.0 (0.0–0.0)	0.730
Drowsiness	0.0 (0.0–33.3)	0.0 (0.0–33.3)	0.0 (0.0–0.0)	0.005*
Hairloss	0.0 (0.0–0.0)	0.0 (0.0–0.0)	0.0 (0.0–0.0)	0.122
Itchy skin	0.0 (0.0–33.3)	0.0 (0.0–33.3)	0.0 (0.0–33.3)	0.260
Weakness of legs	0.0 (0.0–0.0)	0.0 (0.0–0.0)	0.0 (0.0–0.0)	0.063
Bladder control	0.0 (0.0–33.3)	0.0 (0.0–33.3)	0.0 (0.0–33.3)	0.630
HADS				
Anxiety score	4.0 (2.0–7.0)	5.0 (2.0–8.0)	4.0 (1.0–6.0)	0.221
Depression score	2.0 (1.0–6.0)	2.0 (1.0–7.0)	1.0 (1.0–4.0)	0.270

Data are mean (SD), median (IQR), or n (%)

*EORTC QLQ-BN20* European Organization for Research and Treatment of Cancer Quality of Life Questionnaire, Brain Neoplasm Module, *HADS* Hospital Anxiety and Depression Scale, *SF-36* Short-Form Health Survey 36

*P =  ≤ .05 for the comparisons between convexity and skull base cohort and are derived from the Unpaired T-test or Mann–Whitney U test (continuous variables) and Fisher’s exact test or Pearson’s chi-square test (categorical variables)

### Healthcare utilization

#### Primary care

The general practitioner was consulted by 153 patients (80.5%) in the year prior to assessment, with a median of 3.0 visits (IQR 2.0–5.0) (Table 2). The physiotherapist was consulted by 72 (37.9%) patients with a median of 10.0 visits (IQR 5.0–30.0).

**Table 2:** Average healthcare utilization over the past 12 months over the total group, convexity, and skull base meningioma patients

Healthcare service	Total cohort (n = 190)	Visits among those visiting	Convexity meningioma patients (n = 92)	Visits among those visiting	Skull base meningioma patients (n = 93)	Visits among those visiting	*P*-value
Primary care							
General practitioner	153 (80.5%)	3.0 (2.0–5.0)	75 (81.5%)	3.0 (2.0–5.0)	74 (79.6%)	3.0 (1.0–5.0)	0.737
Physiotherapist	72 (37.9%)	10.0 (5.0–30.0)	30 (32.6%)	12.0 (3.5–42.5)	39 (41.9%)	10.0 (6.0–20.0)	0.190
Medical specialist care							
Neurologist	48 (25.4%)	2.0 (1.0–2.0)	23 (25.0%)	2.0 (1.0–2.0)	22 (23.9%)	2.0 (1.0–2.0)	0.864
Neurosurgeon	27 (14.3%)	1.0 (1.0–2.0)	8 (8.7%)	2.0 (1.0–2.0)	18 (19.6%)	1.0 (1.0–1.0)	0.034*
Oncologist	3 (1.6%)	2.0 (1.0–2.0)	2 (2.2%)	1.5 (1.0–2.0)	1 (1.1%)	2.0	1.000
Radiation oncologist	3 (1.6%)	2.0 (1.0 -2.0)	0 (0.0%)	−	3 (3.3%)	2.0 (1.0–2.0)	0.246
Ophthalmologist	39 (20.6%)	1.0 (1.0–2.0)	11 (12.0%)	2.0 (1.0–3.0)	26 (28.3%)	1.0 (1.0–2.0)	0.006*
Dermatologist	11 (5.8%)	1.0 (1.0–3.0)	6 (6.5%)	1.5 (1.0–2.5)	5 (5.4%)	1.0 (1.0–3.5)	0.756
ENT specialist	13 (6.9%)	2.0 (1.5–3.5)	7 (7.6%)	3.0 (2.0–6.0)	5 (5.4%)	2.0 (1.0–2.0)	0.550
Endocrinologist	12 (6.3%)	1.5 (1.0–2.0)	2 (2.2%)	1.0 (1.0–1.0)	9 (9.8%)	2.0 (1.0–2.0)	0.030*
Physiatrist	3 (1.6%)	2.0 (2.0–24.0)	3 (3.3%)	3.0 (2.0–24.0)	0 (0.0%)	−	0.246
Plastic surgeon	3 (1.6%)	3.0 (1.0–10.0)	3 (3.3%)	3.0 (1.0–10.0)	0 (0.0%)	−	0.246
Anesthesiologist	2 (1.1%)	2.0 (1.0–2.0)	0 (0.0%)	−	2 (2.2%)	2.0 (1.0–3.0)	0.497
Radiologist	8 (4.2%)	1.0 (1.0–1.0)	4 (4.3%)	1.0 (1.0–1.0)	4 (4.3%)	1.0 (1.0–1.0)	1.000
Total no. of different specialists							0.835
0	49 (25.8%)	−	25 (27.2%)	−	23 (24.7%)	−	
1	68 (35.8%)	2.0 (1.0–2.0)	32 (34.8%)	2.0 (1.0–2.0)	36 (38.7%)	1.0 (1.0–2.3)	
2	35 (18.4%)	3.0 (3.0–5.0)	19 (20.7%)	4.0 (3.0–6.0)	14 (15.1%)	3.0 (2.8–4.3)	
3	25 (13.2%)	6.0 (3.3–8.0)	11 (12.0%)	6.0 (4.0–9.0)	12 (12.9%)	5.0 (3.0–8.0)	
> 3	12 (6.3%)	10.0 (5.0–15.0)	5 (5.4%)	9.5 (7.0–30.8)	7 (7.5%)	10.0 (5.0–15.0)	
Mental healthcare							
Psychologist	14 (7.4%)	6.0 (1.8–15.0)	8 (8.7%)	4.0 (1.0–12.8)	6 (6.5%)	7.0 (3.8–16.3)	0.564
Psychiatrist	4 (2.1%)	5.5 (1.5–80.0)	3 (3.3%)	8.0 (3.0–104.0)	1 (1.1%)	1.0	0.368
Psychotherapist	2 (1.1%)	11.0 (10.0–12.0)	1 (1.1%)	10.0	1 (1.1%)	12.0	1.000
Hospital admission	22 (11.6%)	1.0 (1.0–1.3)	11 (12.0%)	1.0 (1.0–2.0)	11 (11.8%)	1.0 (1.0–1.0)	0.978
Rehabilitation center	2 (1.1%)	1.0 (1.0–1.0)	2 (2.2%)	1.0 (1.0–1.0)	0 (0.0%)	−	0.243
Emergency department visit	29 (15.3%)	1.0 (1.0–2.0)	9 (9.9%)	1.0 (1.0–2.0)	20 (21.5%)	1.0 (1.0–2.0)	0.031

Data are median (IQR) or n (%)

*ENT* ear, nose, and throat

*P =  ≤ .05 for the comparisons between convexity and skull base cohort and are derived from Fisher’s exact test or Pearson Chi-squared test

#### Medical specialist care

The majority of patients (n = 140, 73.7%) had consulted a medical specialist in the year prior to assessment, most commonly the neurologist (n = 48, 25.4%), followed by the ophthalmologist (n = 39, 20.6%), neurosurgeon (n = 27, 14.3%), and the ear, nose, throat (ENT) specialist (n = 13, 6.9%). Skull base meningioma patients consulted the neurosurgeon, ophthalmologist, and endocrinologist more often than convexity meningioma patients (18 vs 8, 26 vs 11 and 9 vs 2, respectively). The neurologist was consulted by 23 (25.0%) convexity meningioma patients and 22 (23.9%) skull base meningioma patients in the previous year.

#### Mental healthcare

Fourteen patients (7.4%) had visited a psychologist, 4 (2.1%) a psychiatrist, and 2 (1.1%) a psychotherapist in the twelve months prior to assessment. No patients were admitted to a mental healthcare facility.

#### Hospital admissions and emergency care

Twenty-nine patients (15.3%) had visited the emergency department at least once (median 1.0, IQR 1.0–2.0), of which most patients (n = 20, 69.0%) had a skull base meningioma. Reasons for visitation were apparently unrelated to meningiomas (i.e., trauma, infection), except for one patient presenting with seizures and another patient with severe headache and nausea. Furthermore, 22 patients (11.6%) had been admitted to the hospital at least once, with a median duration of one day (median 1.0, IQR 1.0–1.3).

### Determinants of healthcare utilization

Patients treated surgically (OR 0.22, 95% CI 0.06; 0.77), and with a better physical (OR 0.95, 95% CI 0.92; 0.98) and mental HRQoL (OR 0.96, 95% CI 0.93; 0.99) according to the SF-36 had lower specialist care utilization (Table 3). Conversely, patients with visual deficits as presenting symptom (OR 3.37, 95% CI 1.53; 7.42), those who reported more uncertainty of future (OR 1.02, 95% CI 1.01; 1.04) and impaired visual function (OR 1.03, 95% CI 1.01; 1.05) according to the EORTC QLQ-BN20 and those with increased anxiety (OR 1.19, 95% CI 1.09; 1.30) or depression (OR 1.14, 95% CI 1.04; 1.25) according to the HADS had higher specialist care utilization.

**Table 3:** Determinants of medical specialist utilization and healthcare costs among 190 meningioma patients

Determinant	Higher specialist utilization			Healthcare costs		
	OR	95% CI	*P*-value	*β (€)*	95% CI	*P-*value
Tumor size at diagnosis (largest diameter, mm)^1,2^	1.01	0.99; 1.03	0.500	− 4	− 26; 19	0.751
Number of tumors^1,2^	1.01	0.99;1.03	0.310	6	− 18; 30	0.626
Symptoms at presentation (multiple options possible per patient)^1,2^						
Epilepsy	0.60	0.27; 1.35	0.216	406	− 634; 1447	0.442
Motor deficit	0.87	0.33; 2.00	0.738	172	− 910; 1254	0.755
Sensory deficit	1.60	0.62; 4.12	0.326	608	− 541; 1758	0.298
Visual deficit	3.37	1.53; 7.42	0.003*	− 196	− 1069; 677	0.658
Cognitive impairment	0.40	0.13; 1.22	0.108	121	− 1347; 1590	0.871
Psychological impairment	1.49	0.28; 8.00	0.643	− 525	− 2557; 1507	0.611
Headache	1.04	0.47; 2.30	0.925	1341	333; 2349	0.009*
Other	0.93	0.44; 1.97	0.854	− 859	− 1832; 114	0.083
Incidental finding	1.10	0.37; 3.23	0.868	− 556	− 1937; 825	0.428
WHO Grade^1,2^	0.84	0.25; 2.85	0.782	554	− 1150; 2258	0.521
Primary treatment^1,2^						
Surgery	0.22	0.06; 0.77	0.018*	79	− 1105; 1263	0.895
Radiotherapy	5.99	0.74; 48.54	0.094	448	− 1266; 2163	0.606
Conservative treatment	3.41	0.71; 16.30	0.125	− 503	− 2053; 1047	0.523
Reresection (n = 167; treated with primary surgery)	1.93	0.54; 6.92	0.315	2054	435; 3672	0.013*
Charlson Comorbidity Index^1,2,3^	1.30	0.94; 1.79	0.108	210	− 139; 559	0.237
SF-36^1,2,3^						
Physical component score	0.95	0.92; 0.98	0.003*	− 81	− 117; -45	< 0.0001*
Mental component score	0.96	0.93; 0.99	0.008*	− 21	− 58; 15	0.252
EORTC QLQ BN20^1,2,3^						
Uncertainty of future	1.02	1.01; 1.04	0.006*	13	− 5; 31	0.166
Visual dysfunction	1.03	1.01; 1.05	0.005*	18	− 2; 38	0.075
Motor dysfunction	1.02	1.00; 1.04^◊^	0.0.069	34	11; 58	0.004*
Communication deficit	1.01	1.00; 1.03^◊^	0.136	14	− 5; 32	0.149
Headaches	1.00	0.99; 1.02	0.408	22	10; 34	< 0.001*
Seizures	0.99	0.96; 1.02	0.487	18	− 16; 51	0.307
Drowsiness	1.01	1.00; 1.03^◊^	0.116	19	4; 33	0.012*
Hair loss	1.01	0.99; 1.02	0.450	0	− 20; 21	0.971
Itchy skin	1.01	1.00; 1.03^◊^	0.056	21	6; 37	0.006*
Leg weakness	1.00	0.99; 1.02	0.564	25	7; 44	0.007*
Bladder control	1.01	1.00; 1.03^◊^	0.122	19	2; 35	0.024*
HADS^1,2,3^						
Anxiety score	1.19	1.09; 1.30	< 0.001*	47	− 47; 141	0.328
Depression score	1.14	1.04; 1.25	0.005*	60	− 36; 156	0.222

*EORTC QLQ-BN20* European Organization for Research and Treatment of Cancer Quality of Life Questionnaire, Brain Neoplasm Module, *HADS* Hospital Anxiety and Depression Scale, *SF-36* Short-Form Health Survey 36

*P =  ≤ .05 for the comparisons between convexity and skull base cohort and are derived from linear or logistic regression analysis

^◊^Value of 1.00 in the 95% CI due to rounding. 1,2,3 Adjusted for age (1), sex (2), and education (3)

### Healthcare costs

**Fig. 1:**
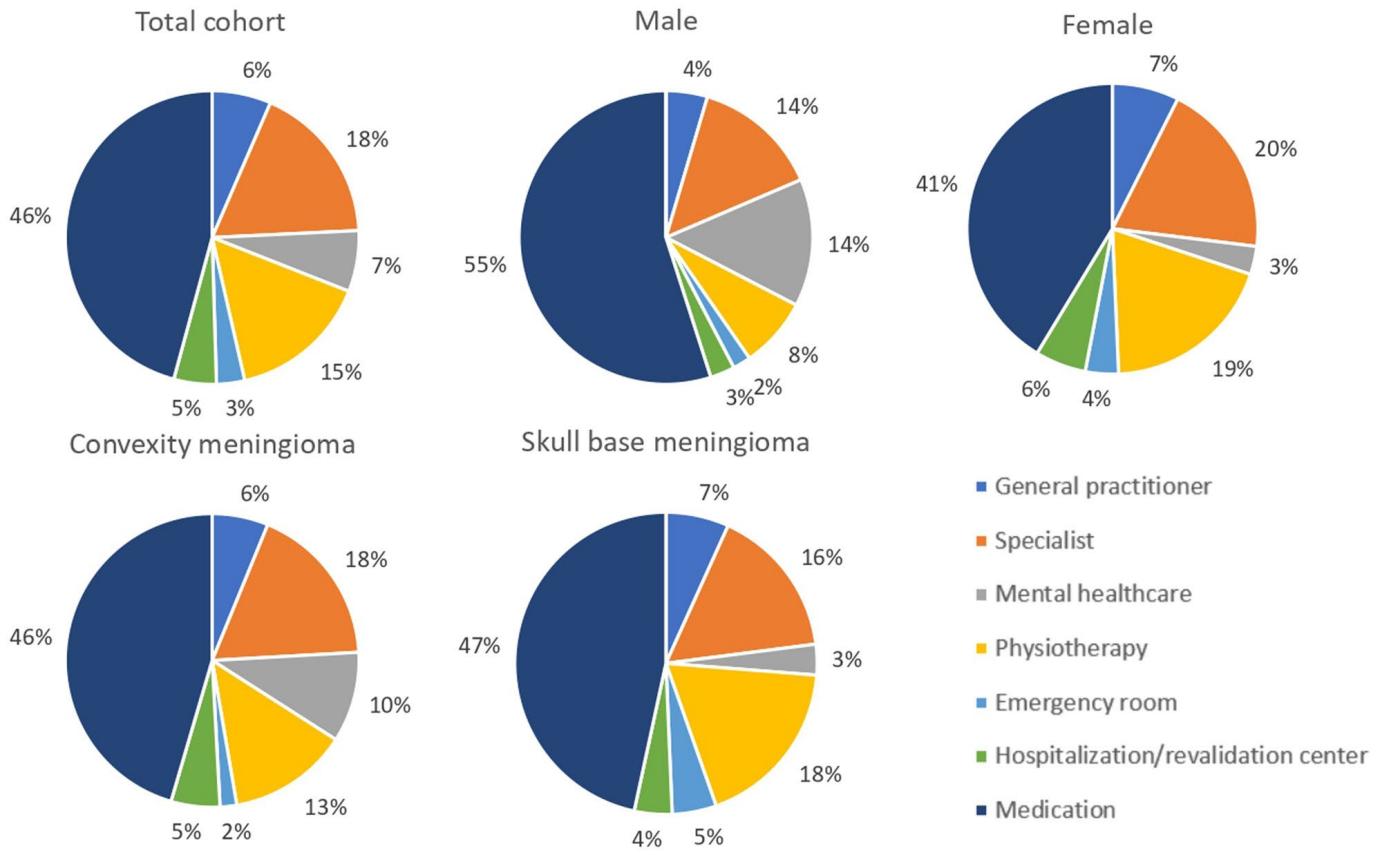
Pie charts presenting the proportions of healthcare costs for the total cohort and categorized by sex and tumor location

Median annual healthcare costs of meningioma patients who had used healthcare in the year prior to assessment were € 871 (IQR 262–1933) (Table 4). Medication constituted the largest expenditure (45.8% of all costs), followed by specialist care (17.7%) and physiotherapy (15.5%) (Fig. 1). Anti-seizure medication constituted the largest medication costs (median 1986, IQR 1169–3294).

**Table 4:** Healthcare costs in euro (€) on average in the 12 months prior to the study among 190 patients with intracranial meningioma, presented for the total cohort, convexity meningioma, and skull base meningioma patients

Medical costs	Total cohort (n = 190)	Costs among those visiting in euros	Convexity meningioma patients (n = 92)	Costs among those visiting in euros	Skull base meningioma patients (n = 93)	Costs among those visiting in euros	*P*-value
Primary care							
General practitioner	153 (80.5%)	99.00 (66.00–165.00)	75 (81.5%)	99.00 (66.00–165.00)	74 (79.6%)	99.00 (33.00–165.00)	0.531
Physiotherapist	72 (37.9%)	330.00 (165.00–990.00)	30 (32.6%)	396.00 (115.50–1402.50)	39 (41.9%)	330.00 (198.00–660.00)	0.855
Medical specialist							
Neurologist	48 (25.4%)	163.00 (100.00–326.00)	23 (25.0%)	163.0 0 (80.00–326.00)	22 (23.9%)	326.00 (142.25–326.00)	0.390
Neurosurgeon	27 (14.3%)	163.00 (163.00–326.00)	8 (8.7%)	244.50 (160.75–326.00)	18 (19.6%)	163.00 (142.25–163.00)	0.261
Oncologist	3 (1.6%)	326.00 (163.00–326.00)	2 (2.2%)	244.50 (163.00–326.00)	1 (1.1%)	326.00	†
Radiation oncologist	3 (1.6%)	326.00 (163.00–326.00)	0 (0.0%)	−	3 (3.3%)	326.00 (163.00–326.00)	†
Ophthalmologist	39 (20.6%)	163.00 (163.00–326.00)	11 (12.0%)	240.00 (163.00–326.00)	26 (28.3%)	163.00 (162.25–326.00)	0.589
Dermatologist	11 (5.8%)	163.00 (80.00–489.00)	6 (6.5%)	244.50 (80.00–407.50)	5 (5.4%)	163.00 (121.50–570.50)	0.792
ENT specialist	13 (6.9%)	326.00 (163.00–403.00)	7 (7.6%)	326.00 (240.00–652.00)	5 (5.4%)	163.00 (161.50–326.00)	0.149
Endocrinologist	12 (6.3%)	163.00 (163.00–326.00)	2 (2.2%)	163.00 (163.00–163.00)	9 (9.8%)	163.00 (163.00–326.00)	0.582
Physiatrist	3 (1.6%)	326.00 (240.00–3912.00)	3 (3.3%)	326.00 (240.00–3912.00)	0 (0.0%)	−	†
Plastic surgeon	3 (1.6%)	489.00 (163.00–1630.00)	3 (3.3%)	486.00 (163.00–1630.00)	0 (0.0%)	−	†
Anesthesiologist	2 (1.1%)	201.50 (163.00–240.00)	0 (0.0%)	−	2 (2.2%)	201.50 (163.00–240.00)	†
Radiologist	8 (4.2%)	163.00 (163.00–163.00)	4 (4.3%)	163.00 (100.75–163.00)	4 (4.3%)	163.00 (163.00 -163.00)	0.686
Mental healthcare							
Psychologist	14 (7.4%)	384.00 (112.00–960.00)	8 (8.7%)	256.00 (64.00–816.00)	6 (6.5%)	448.00 (240.00–1040.00)	0.268
Psychiatrist	4 (2.1%)	517.00 (141.00–7520.00)	3 (3.3%)	752.00 (282.00–9776.00)	1 (1.1%)	94.00	†
Psychotherapist	2 (1.1%)	1034.00 (940.00–1128.00)	1 (1.1%)	940.00	1 (1.1%)	1128.00	†
Hospital admission	22 (11.6%)	443.00 (443.00–752.75)	11 (12.0%)	443.00 (443.00–1284.00)	11 (11.8%)	443.00 (443.00–443.00)	0.153
Rehabilitation center	2 (1.1%)	460.00 (460.00–460.00)	2 (2.2%)	460.00 (460.00–460.00)	0 (0.0%)	−	†
Emergency department visit	29 (15.3%)	259.00 (259.00–518.00)	9 (9.9%)	259.00 (259.00–518.00)	20 (21.5%)	259.00 (259.00–518.00)	0.717
Total medical costs	173 (91.1%)	540.00 (226.00–1181.00)	84 (91.3%)	443.50 (160.75–1178.50)	85 (91.4%)	625.00 (327.50–1160.50)	0.221
Medication costs							
Carbamazepine	11 (3.4%)	1168.80 (1168.80–2337.60)	5 (5.4%)	1753.20 (1168.80–2629.80)	6 (6.5%)	1168.80 (474.83–2337.60)	0.258
Clobazam	3 (1.6%)	47.48 (13.57–47.48)	2 (2.2%)	30.52 (13.57–47.48)	1 (1.1%)	47.48	†
Clonazepam	2 (1.1%)	602.66 (241.07–964.23)	1 (1.1%)	241.07	0 (0.0%)	−	†
Valproic acid	9 (4.7%)	1558.40 (1168.80–3506.40)	6 (6.5%)	1480.48 (1168.80–2922.00)	3 (3.2%)	2337.60 (1168.80–7480.32)	0.510
Phentoin	1 (0.5%)	1661.89	1 (1.1%)	1661.89	0 (0.0%)	−	†
Gabapentine	3 (1.6%)	3155.76 (1051.92–9467.28)	0 (0.0%)	−	2 (2.2%)	6311.52 (3155.76–9467.28)	†
Lamotrigine	8 (4.2%)	6574.50 (3287.25–12,491.55)	5 (5.4%)	5259.60 (3944.70–13,149.00)	3 (3.2%)	7889.40 (2629.80–10,519.20)	0.761
Levetiracetam	14 (7.4%)	803.55 (401.78–903.99)	9 (9.8%)	803.55 (502.22–1205.33)	5 (5.4%)	803.55 (301.33–803.55)	0.264
Oxcarbazepine	2 (1.1%)	558.83 (372.56–745.11)	1 (1.1%)	372.56	1 (1.1%)	745.11	†
Pregabaline	1 (0.5%)	540.57	1 (1.1%)	540.57	0 (0.0%)	−	†
Topiramate	1 (0.5%)	1168.80	1 (1.1%)	1168.80	0 (0.0%)	−	†
Total costs of anti-seizure medication	41 (21.6%)	1985.92 (1168.80–3294.56)	23 (25.0%)	1985.92 (1176.11–3433.35)	17 (18.3%)	1548.66 (803.55–5341.78)	0.443
Diazepam	2 (1.1%)	147.93 (131.49–164.36)	1 (1.1%)	164.36	1 (1.1%)	131.49	†
Flunitrazepam	1 (0.5%)	153.41	1 (1.1%)	153.41	0 (0.0%)	−	†
Nitrazepam	1 (0.5%)	191.76	0 (0.0%)	−	1 (1.1%)	191.76	†
Oxazepam	3 (1.6%)	80.36 (5.74–200.89)	2 (2.2%)	140.62 (80.36–200.89)	1 (1.1%)	5.74	†
Temazepam	5 (2.6%)	20.09 (11.48–80.36)	3 (3.3%)	80.36 (20.09–80.36)	2 (2.2%)	11.48 (2.87–20.09)	†
Zopiclon	1 (0.5%)	6.26	1 (1.1%)	6.26	0 (0.0%)	−	†
Total costs of benzodiazepines	11 (3.4%)	80.36 (6.26–191.76)	6 (6.5%)	122.36 (16.63–245.63)	5 (5.4%)	20.09 (4.30–161.62)	0.234
Venlafaxine	1 (0.5%)	98.62	1 (1.1%)	98.62	0 (0.0%)	−	†
Citalopram	1 (0.5%)	120.53	1 (1.1%)	120.53	0 (0.0%)	−	†
Escitalopram	1 (0.5%)	29.22	1 (1.1%)	29.22	0 (0.0%)	−	†
Fluoxetine	1 (0.5%)	18.26	1 (1.1%)	18.26	0 (0.0%)	−	†
Paroxetine	9 (4.7%)	116.88 (58.44–204.54)	6 (6.5%)	116.88 (58.44–146.10)	3 (3.2%)	175.32 (58.44–233.76)	0.502
Trazodon	1 (0.5%)	152.31	1 (1.1%)	152.31	0 (0.0%)	−	†
Amitryptiline	3 (1.6%)	80.36 (80.36–602.66)	2 (2.2%)	341.51 (80.36–602.66)	1 (1.1%)	80.36	†
Clomipramine	1 (0.5%)	1369.69	0 (0.0%)	−	1 (1.1%)	1369.69	†
Mirtazepine	2 (1.1%)	18.26 (18.26–18.26)	0 (0.0%)	−	2 (2.2%)	18.26 (18.26–18.26)	†
Total costs of antidepressants	18 (9.5%)	113.23 (58.44–233.76)	11 (12.0%)	116.88 (58.44–233.76)	7 (7.3%)	80.36 (18.26–233.76)	0.649
Hydrocortisone	3 (1.6%)	2220.72 (2220.72–3331.08)	0 (0.0%)	−	3 (3.2%)	2220.72 (2220.72–3331.08)	†
Prednisolone	3 (1.6%)	16.44 (10.96–38.35)	1 (1.1%)	16.44	2 (2.2%)	24.65 (10.96–38.35)	†
Tibolone	2 (1.1%)	178.97 (178.97–178.97)	1 (1.1%)	178.97	1 (1.1%)	178.97	†
Levothyroxine	16 (8.4%)	219.15 (192.85–219.15)	5 (5.4%)	219.15 (219.15–383.51)	11 (11.8%)	219.15 (109.58–219.15)	0.162
Total costs of hormone replacement therapy	21 (11.1%)	219.15 (109.58–369.27)	7 (7.6%)	219.15 (178.97–219.15)	14 (15.1%)	219.15 (109.58–973.21)	0.909
Total medication costs	75 (39.5%)	1168.80 (146.10–2337.60)	37 (40.2%)	1387.95 (191.76–2337.60)	37 (39.8%)	693.98 (120.53–2375. 59)	0.352
Overall costs		871.08 (262.00–1933.10)		804.50 (168.50–2132.10)		871.15 (271.69–1727.50)	0.812

Data are median (IQR) or n (%)

*ENT* ear, nose, and throat

^†^P-value could not be calculated, because the number of patients in the cohorts were too low

^*^P =  ≤ .05 for the comparisons between convexity and skull base cohort and are derived from the Mann–Whitney U test

Median annual healthcare costs were not significantly higher for skull base meningioma patients compared with convexity meningioma patients (€ 871, IQR 272–1728 vs € 805, IQR 169–2132), nor did any other costs.

### Determinants of healthcare costs

Patients with better physical functioning (β (€) = − 82, 95% CI − 117; − 45) measured with the SF-36 had lower healthcare costs; in other words, patients with one point higher on the physical component score of the SF-36 had on average €82 lower healthcare costs. Conversely, healthcare costs were higher for patients presenting with headache (β (€) = 1341, 95% CI 333; 2349) and those receiving reresection (β (€) = 2054, 95% CI 435; 3672). Healthcare costs were also higher for patients who reported more motor dysfunction (β (€) = 34, 95% CI 11; 58), complaints of headaches (β (€) = 22, 95% CI 10; 34), drowsiness (β (€) = 19, 95% CI 4; 33), itchy skin (β (€) = 21, 95% CI 6; 37), leg weakness (β (€) = 25, 95% CI 7; 44) and impairments with bladder control (β (€) = 19, 95% CI 2; 35) as measured with the EORTC QLQ-BN20.

### Impact of anxiety and depression

A subanalysis between patients with a HADS anxiety *or* depression score > 7 and ≤ 7 did not show a significant difference in healthcare costs (Supplemental Tables 1–2). However, patients with a HADS anxiety *and* depression score > 7 utilized significantly more mental healthcare compared to patients with a score ≤ 7 (25.6% vs 5.4%; 21.9% vs 7.7%). Both mental component score and physical component score of the SF-36 were significantly lower in patients with a HADS anxiety (MCS: 37.2 vs 53.6; PCS: 41.4 vs 48.9) or depression score > 7 (MCS: 34.2 vs 53.0; PCS: 39.4 vs 48.8). The mean difference of mental and physical HRQoL scores between the groups surpassed the minimal clinically important difference.

## Discussion

This is the first study to investigate healthcare utilization and costs for intracranial meningioma patients during long-term surveillance and to identify their determinants. Main findings of this study are that (1) medication costs constituted the largest expenditure of total annual healthcare costs and that (2) better HRQoL is associated with lower healthcare utilization and costs. Unexpectedly, healthcare utilization and costs did not differ between convexity and skull base meningioma patients during long-term surveillance.

In our study, medication costs constituted the largest expenditure of total healthcare costs and costs of anti-seizure medication were the highest out of all medication costs. Epilepsy is a common first presentation in slow-growing brain tumors, such as meningiomas [12, 13]. More than 25% of meningioma patients present with epilepsy [12–14]. Although seizure freedom is achieved in more than 60% of patients after surgery, approximately 20% of meningioma patients develop seizures postoperatively [14]. Previously, the use of anti-seizure medication in meningioma patients was found to be increased even two years postoperatively compared to healthy controls [15]. Studies have shown that anti-seizure medication use in long-term management of meningioma patients is associated with worse neurocognitive functioning and HRQoL [16, 17]. In addition to the high costs of anti-seizure medication, this raises the question of whether we should focus more on tapering off anti-seizure medication in long-term follow-up.

Interestingly, surgery as primary treatment was associated with low healthcare utilization. Current literature reports improved HRQoL in meningioma patients after surgery, but still lower compared to healthy controls [5, 18, 19]. In a previous study we reported that patients treated with solely surgery seem to report better HRQoL and neurocognitive functioning compared to patients treated primarily with radiotherapy, adjuvant radiotherapy, or reresection [5, 20]. Radiotherapy is usually reserved for patients suffering from comorbidities, patients with anatomically complex tumors, or patients with higher-grade meningiomas [5, 20]. Although treatment choices are made by the situation at diagnosis and some patients are inoperable, we find a better outcome for healthcare utilization in addition to better HRQoL in patients with a single surgical procedure.

This study was not designed to make comparisons with other conditions. Since we focused on meningioma-related costs, we cannot draw conclusions on total costs including unrelated healthcare costs. As a reference the mean general annual healthcare cost of the general Dutch population in 2017 were € 5656 per capita [21]. No previous studies were available for comparison of healthcare utilization and costs in meningioma patients. We believe that our results of determinants of healthcare utilization and costs are reproducible in other countries and healthcare systems (e.g., the correlation of lower HRQoL and higher healthcare utilization and costs). Especially since we have only considered long-term follow-up costs, not active treatment costs. Furthermore, by providing mean visits per patient, comparisons between healthcare systems can be made.

As expected, HRQoL is related to healthcare utilization and costs in meningioma patients, as is known for other conditions [22, 23]. Our findings are in accordance with previous studies on healthcare utilization and costs of brain tumors, where better HRQoL according to the SF-36 was related to lower healthcare utilization and costs [22, 23]. We also related healthcare costs and utilization to the severity of symptoms caused by brain tumors using the EORTC QLQ-BN20.

Continued improvement in surgical and radiotherapeutic techniques for meningioma treatment has increased the number of long-term survivors and patients are often considered cured without complications [5, 18, 19, 24]. However, meningioma patients still show lower levels of HRQoL and impaired neurocognitive functioning and suffer from anxiety and depression on the long-term [5, 18, 19, 24]. It is tempting to speculate that strategies that improve long-term HRQoL in meningiomas (e.g., rehabilitation and addressing unmet needs in psychosocial support early after treatment) could be beneficial in reducing disease burden and improving functional outcome, consequently, reducing long-term healthcare utilization and costs.

Surprisingly, overall healthcare utilization and costs did not differ between convexity and skull base meningioma patients during long-term follow-up, even though higher utilization of the ophthalmologist, neurosurgeon, and ENT specialist was found in skull base meningioma patients. Previous studies on meningioma patients report poor neurocognitive outcomes and worse HRQoL especially in skull base meningioma patients after surgical treatment [17, 25, 26]. Higher healthcare utilization and costs would therefore be expected in skull base meningioma patients. Our study shows that the multidisciplinary approach in long-term surveillance of skull base meningioma patients does not necessarily translate to higher overall annual healthcare costs.

Our previous study reported that meningioma patients had an increased risk of anxiety or depression after a median follow-up of nine years [5]. This study shows that those patients with self-reported anxiety or depression also report a lower mental and physical HRQoL compared to patients without anxiety or depression. In analogy with studies in other populations, patients with self-reported anxiety or depression utilize more specialist care compared to patients without, while overall healthcare costs do not differ between groups [27, 28]. Noteworthy is that although patients with self-reported symptoms of anxiety and depression utilize more mental healthcare, most patients (anxious 75.0%; depressed 78.1%) do not utilize mental healthcare at all. Low utilization of mental healthcare despite lower mental and physical HRQoL may indicate underrecognition of long-term disease burden and inadequate long-term care for these patients.

Main limitations of our study lie in the observational cross-sectional design. Therefore, no conclusions can be drawn on possible improvement or deterioration of outcomes after treatment and results might be affected by confounding and other types of bias. Due to the retrospective collection of data, we might have missed certain poorly reported perioperative complications that may also impact future functioning and well-being. To reduce the impact of confounding, we corrected our analyses for multiple known and measured confounders using multivariable analyses. Furthermore, we might miss meningioma-specific HRQoL issues as we used the general SF-36 questionnaire and the brain cancer-specific EORTC QLQ-BN20. There are currently no validated meningioma-specific HRQoL questionnaires available [29]. Furthermore, the study-specific healthcare utilization questionnaire, which is not validated, did not contain more specific questions about meningioma-related care (e.g., reresection, medical imaging for long-term follow-up) and therefore other relevant meningioma-care-related costs may not have been taken into account, while meningioma-care-unrelated cost might have been included in the analysis. Lastly, although self-reported data is the preferred method of assessing healthcare costs [30], this also imposes a risk of recall bias, particularly with a timeframe of one year.

## Conclusions

Medication costs constituted the largest expenditure of total healthcare costs, of which anti-seizure medication are the main contributor. Lower patient-reported HRQoL is related to higher healthcare utilization and costs of treated intracranial WHO grade I or II meningioma patients. To improve long-term functional outcome and level of HRQoL, additional care and support could be considered in patients with impaired mental health and those with high disease burden. We identified some patient, tumor, and treatment characteristics that are determinants for healthcare costs and utilization, although these are probably not amendable to change in this heterogeneous disease with individualized treatment decision making. However, better insights in these factors may identify those cases with higher need for support and care.

## Supplementary Information

Below is the link to the electronic supplementary material.

Supplementary file1 (DOCX 34 kb)

## Data Availability

The datasets generated during and/or analyzed during the current study are available from the corresponding author on reasonable request.
