# Correction to: Healthcare utilization and costs among intracranial meningioma patients during long-term follow-up

**DOI:** 10.1007/s11060-023-04255-0

**Published:** 2023-02-13

**Authors:** Kevin A. Huynh, Eva C. Coopmans, Amir H. Zamanipoor Najafabadi, Linda Dirven, Saskia M. Peerdeman, Nienke R. Biermasz, Marco J. T. Verstegen, Wouter R. van Furth, the Dutch Meningioma Consortium, Florien W. Boele, Martin Klein, Johan Koekkoek, Frank Lagerwaard, Pim B. van der Meer, Martin J. B. Taphoorn, Wouter A. Moojen, Jaap C. Reijneveld

**Affiliations:** 1 Division of Endocrinology, Department of Medicine, Leiden University Medical Center, Albinusdreef 2, 2333 ZA Leiden, The Netherlands; 2 Center for Endocrine Tumors Leiden (CETL), Center for Pituitary Care, Leiden University Medical Center, Leiden, The Netherlands; 3 Department of Neurosurgery, University Neurosurgical Center Holland, Haaglanden Medical Center, and the Hague Teaching Hospitals, Leiden University Medical Center, Leiden, The Netherlands; 4 Department of Neurology, Leiden University Medical Center, Leiden, The Netherlands; 5 Department of Neurology, Haaglanden Medical Center, The Hague, The Netherlands; 6 Department of Neurosurgery, Amsterdam University Medical Centers, Location VUmc, Amsterdam, The Netherlands

**Correction to: Journal of Neuro-Oncology** 10.1007/s11060-022-04223-0

In the initial online publication, the name of author Amir H. Zamanipoor Najafabadi was incorrectly specified as Amir. H.Z. Najafabadi. The original article has been corrected.

