# Supplementary material for: Healthcare utilization and costs among intracranial meningioma patients during long-term follow-up
Source: J Neurooncol. 2023 Jan 10;161(2):357–70. doi: 10.1007/s11060-022-04223-0 (PMC9988802; doi:10.1007/s11060-022-04223-0)
Supplement: Supplementary file 1 — Supplementary file1 (DOCX 34 kb) [file 11060_2022_4223_MOESM1_ESM.docx]

**Supplemental File**

**Title: Healthcare Utilization and Costs among Intracranial Meningioma Patients during Long-Term Follow-Up**

**Authors:** Kevin A. Huynh^1,2,3*^, BSc, Eva C. Coopmans^1,2,3*^, MD, PhD, Amir H. Zamanipoor Najafabadi^3,4^, MD, PhD, Linda Dirven^4,5^, PhD, Saskia M. Peerdeman^6^, MD, PhD, Nienke R. Biermasz^1,2^, MD, PhD, Marco J.T. Verstegen^3^, MD, Wouter R. van Furth^3^, MD, PhD, on behalf of the Dutch Meningioma Consortium.

*These authors contributed equally

**Affiliations:**

^1^Department of Medicine, Division of Endocrinology, Leiden University Medical Center, Leiden, The Netherlands.

^2^Center for Endocrine Tumors Leiden (CETL), Center for Pituitary Care, Leiden University Medical Center, Leiden, The Netherlands.

^3^University Neurosurgical Center Holland, Department of Neurosurgery, Leiden University Medical Center, Haaglanden Medical Center, and the Hague Teaching Hospitals, The Netherlands.

^4^Department of Neurology, Leiden University Medical Center, Leiden, The Netherlands.

^5^Department of Neurology, Haaglanden Medical Center, The Hague, The Netherlands.

^6^Department of Neurosurgery, Amsterdam University Medical Centers, location VUmc, Amsterdam, the Netherlands.

**ORCiD ID:**

KAH – 0000-0002-7137-1074

ECC – 0000-0003-4780-2468

AHZN – 0000-0003-2400-2070

LD – 0000-0001-9157-9895

SMP – 0000-0001-5691-3210

NRB – 0000-0001-5817-3594

MJTV – 0000-0001-9739-7515

WRvF – 0000-0001-5208-921X

**Corresponding Author’s name and current institution:** Eva C. Coopmans. Department of Medicine, Division of Endocrinology, Leiden University Medical Center, Leiden, The Netherlands. Center for Endocrine Tumors Leiden (CETL), Center for Pituitary Care, Leiden University Medical Center, Leiden, The Netherlands. University Neurosurgical Center Holland, Leiden University Medical Center, Haaglanden Medical Center and Haga Teaching Hospital, Leiden and The Hague, the Netherlands. Department of Neurology, Leiden University Medical Center, Leiden, the Netherlands

**Address:** Albinusdreef 2, 2333ZA, Leiden, The Netherlands

**Corresponding Author’s Email:** [e.c.coopmans@lumc.nl](mailto:e.c.coopmans@lumc.nl)

**Health-Related Quality of Life and Patient-Reported Anxiety and Depression**

The SF-36 is a validated HRQoL questionnaire consisting of 36 items encompassing 8 domains: physical functioning, physical role functioning, bodily pain, general health, vitality, social functioning, emotional role, and mental health. These subscales can also be scored from 0 to 100 in a physical component score (PCS) and mental component score (MCS). A higher score indicates a better HRQoL[1]. Minimal clinically important differences (MCIDs) were set at 4.6 points for the MCS and 3.0 points for the PCS in accordance to previous studies[2].

The EORTC QLQ-BN20 is a validated module for the assessment of HRQoL issues for patients with a primary brain tumor. It consists of 4 multi-item scales: uncertainty of future (4 items), visual dysfunction (3 items), motor dysfunction (3 items), and communication deficit (3 items). Next to that, 7 single items are assessed: headaches, seizures, drowsiness, hair loss, itchy skin, leg weakness, and bladder control. All scales and items are scored in a range of 0 to 100. A higher score meaning more severe symptoms[3].

The HADS is a validated scale for detection of anxiety and depression. It consists of 1 scale for anxiety and 1 scale for depression. Each scale consists of 7 questions on emotion and behavior in the previous week that can be scored to a maximum of 4 points. Scores for each scale range from 0 to 21. A higher score indicates a higher likeliness of anxiety or depression[4]. In the supplemental subanalysis patients were divided into a group with a score >7 and a group with a score ≤7 in accordance with the classifications in the validated scale (i.e., 0-7 normal, 8-11 possible anxiety/depression, 11-21 highly probable anxiety/depression)[4].

The SF-36, EORTC QLQ-BN20 and HADS have been validated previously in brain tumor patients, of which HADS was only validated in glioma patients (i.e., not in meningioma patients)[5-8].

**Missing values**

Missing values of determinants as percentage of the whole cohort: education level 2.6%; number of tumors 3.7%; WHO grade 4.2% (surgically treated patients); CCI 1.6%; physical component score 0.5%; mental component score 1.1%; EORTC QLQ-BN20 scores of uncertainty of future 2.6%, visual disfunction 0.5%, motor disfunction 1.6%, headaches 0.5%, seizures 0.5%, bladder control 0.5%; HADS depression score 1.6%. The determinants age at diagnosis, sex, symptoms at presentation, primary treatment, time since treatment, EORTC QLQ-BN20 scores of communicative deficit, drowsiness, hair loss, itchy skin, weakness of legs, and HADS anxiety score had no missing data.

**Population Characteristics Stratified by Tumor Location**

Stratified by tumor location, patients with skull base meningioma (n = 93) were more often female (86.0% vs 70.7%) and harbored a smaller tumor at diagnosis (median 37.0 mm vs 40.5 mm) when compared with convexity meningioma patients (n = 92) (Table 1). Patients with skull base meningioma presented more often with visual and sensory deficits than patients with convexity meningioma (41.9% vs 8.7% and 18.3% vs 7.6%, respectively). In contrast, patients with convexity meningioma were more likely to present with epilepsy (21.7% vs 10.8%), motor deficit (20.7% vs 9.7%), cognitive impairment (12.0% vs 3.2%), and as an incidental finding (14.1% vs 4.3%). Skull base meningioma patients underwent adjuvant radiotherapy more often than convexity meningioma patients (21.5% vs 6.5%, p = 0.003). Surgically treated skull base meningioma patients suffered more often from cerebrospinal fluid leak (8 vs 0, p = 0.007). Skull base and convexity meningioma patients had similar scores on 9/11 EORTC QLQ-BN20 scales, except for lower motor dysfunction (Δ: -11.1, p = 0.012) in skull base meningioma patients. No differences between the two subgroups were observed for SF-36 and HADS.

**Supplemental Table 1. Subanalysis of intracranial meningioma patients during long-term follow-up stratified by HADS anxiety score**

|  | HADS anxiety >7  (n = 43) | HADS anxiety ≤ 7  (n = 147) | *P*-value |
| --- | --- | --- | --- |
| Medical costs | 540.00 (132.00 – 1310.00) | 460.00 (113.00 – 1094.00) | 0.532 |
| Medication costs | 0.00 (0.00 – 493.09) | 0.00 (0.00 – 219.15) | 0.277 |
| Total costs | 1222.00 (165.00 – 1917.73) | 817.00 (262.00 – 1979.20) | 0.296 |
| Specialist utilization (missing n = 3) |  |  | 0.038* |
| High (≥3 visits) | 32 (76.2) | 85 (58.6) |  |
| Low (<3 visits) | 10 (23.8) | 60 (41.4) |  |
| Mental healthcare utilization | 11 (25.6) | 8 (5.4) | <0.001* |
| SF-36 (missing n = 3) |  |  |  |
| MCS | 37.2 (10.9) | 53.6 (7.4) | <0.0001* |
| PCS | 41.4 (10.4) | 48.9 (10.2) | <0.0001* |

*Data are mean (SD) or n (%). *: P = ≤.05 for the comparisons between convexity and skull base cohort and are derived from the Unpaired T-test (continuous variables) and Fisher’s exact test or Pearson’s chi-square test (categorical variables). Abbreviations: HADS, Hospital Anxiety and Depression Scale; SF-36, Short-Form Health Survey 36.*

**Supplemental Table 2. Subanalysis of intracranial meningioma patients during long-term follow-up stratified by HADS depression score**

|  | HADS depression >7  (n = 32) | HADS depression ≤ 7  (n = 155) | *P*-value |
| --- | --- | --- | --- |
| Medical costs | 682.50 (311.00 – 1288.00) | 405.00 (113.00 – 1150.00) | 0.221 |
| Medication costs | 37.44 (0.00 – 671.15) | 0.00 (0.00 – 219.15) | 0.081 |
| Total costs | 1238.88 (608.27 – 1887.00) | 817.00 (165.00 – 2046.40) | 0.061 |
| Specialist utilization (missing n = 6) |  |  | 0.022* |
| High (≥3 visits) | 25 (80.6) | 90 (58.8) |  |
| Low (<3 visits) | 6 (19.4) | 63 (41.2) |  |
| Mental healthcare utilization | 7 (21.9) | 12 (7.7) | 0.025* |
| SF-36 (missing n = 2) |  |  |  |
| MCS | 34.2 (10.8) | 53.0 (7.5) | <0.0001* |
| PCS | 39.4 (11.3) | 48.8 (9.9) | <0.0001* |

*Data are mean (SD) or n (%). *: P = ≤.05 for the comparisons between convexity and skull base cohort and are derived from the Unpaired T-test (continuous variables) and Fisher’s exact test or Pearson’s chi-square test (categorical variables). Abbreviations: HADS, Hospital Anxiety and Depression Scale; SF-36, Short-Form Health Survey 36.*

**References**

1. Ware JEJ (1992) The MOS 36-ltem Short-Form Health Survey (SF-36): I. Conceptual Framework and Item Selection. Med Care. pp 473-483

2. Edelvik A, Taft C, Ekstedt G, Malmgren K (2017) Health-related quality of life and emotional well-being after epilepsy surgery: A prospective, controlled, long-term follow-up. Epilepsia 58: 1706-1715 doi:<https://doi.org/10.1111/epi.13874>

3. Taphoorn MJB, Claassens L, Aaronson NK, Coens C, Mauer M, Osoba D, Stupp R, Mirimanoff RO, Bent MJvd, Bottomley A (2010) An international validation study of the EORTC brain cancer module (EORTC QLQ-BN20) for assessing health-related quality of life and symptoms in brain cancer patients. European Journal of Cancer. Elsevier, pp 1033-1040

4. Zigmond AS, Snaith RP (1983) The hospital anxiety and depression scale. Acta Psychiatr Scand 67: 361-370 doi:10.1111/j.1600-0447.1983.tb09716.x

5. Bunevicius A (2017) Reliability and validity of the SF-36 Health Survey Questionnaire in patients with brain tumors: a cross-sectional study. Health Qual Life Outcomes 15: 92 doi:10.1186/s12955-017-0665-1

6. Taphoorn MJ, Claassens L, Aaronson NK, Coens C, Mauer M, Osoba D, Stupp R, Mirimanoff RO, van den Bent MJ, Bottomley A (2010) An international validation study of the EORTC brain cancer module (EORTC QLQ-BN20) for assessing health-related quality of life and symptoms in brain cancer patients. Eur J Cancer 46: 1033-1040 doi:10.1016/j.ejca.2010.01.012

7. Rooney AG, McNamara S, Mackinnon M, Fraser M, Rampling R, Carson A, Grant R (2013) Screening for major depressive disorder in adults with cerebral glioma: an initial validation of 3 self-report instruments. Neuro Oncol 15: 122-129 doi:10.1093/neuonc/nos282

8. Bjelland I, Dahl AA, Haug TT, Neckelmann D (2002) The validity of the Hospital Anxiety and Depression Scale. An updated literature review. J Psychosom Res 52: 69-77 doi:10.1016/s0022-3999(01)00296-3
